# HPV-specific risk assessment of cervical cytological abnormalities

**DOI:** 10.1186/s12885-021-08703-w

**Published:** 2021-08-24

**Authors:** Guanglei Zhong, Yuhan Wang, Qingsheng Xie, Rongchun Lin, Tingting Yao

**Affiliations:** 1 Department of Gynecological Oncology, Sun Yat-Sen Memorial Hospital, Sun Yat-Sen University, 107 Yan Jiang West Road, Guangzhou, People’s Republic of China; 2 Key Laboratory of malignant tumor gene regulation and target therapy of Guangdong Higher Education Institutes, Sun Yat-Sen University, Guangzhou, People’s Republic of China

**Keywords:** Cervical cancer, HPV, Cytology

## Abstract

**Background:**

Cytology and HPV genotype screening play an important role in cervical cancer detection. Whether multiple HPV genotyping can predict cytological lesions remains to be further studied.

**Methods:**

Two thousand two hundred twenty-four females were analyzed for cytology and HPV genotypes test. The possibility of predicting cytological lesions by HPV genotypes test was evaluated by multivariate logistic regression and area under the receiver operator characteristic curve (AUC).

**Result:**

Abnormal cytological results were found in 479 participants. A total of 688 patients were detected with HPV infection, 619 with HR-HPV infection and 112 with LR-HRV infection. HPV-52 was found to be the most common type among these patients, and a relatively higher risk of cervical lesions was found in HPV positive females. HPV-16, 31, 33 and 58 were found to have significantly higher infection rates in patients with HSIL and higher lesions. The prediction model was developed based on age and HPV-specific genotypes, with the AUC of 0.73 for cytological abnormalities and 0.82 for HSIL and higher lesions.

**Conclusion:**

HPV-16, 31, 33 and 58 infection are significant risk factors for cervical lesions. Combined HPV genotypes test can effectively predict cytological abnormalities.

## Introduction

Cervical cancer remains one of the most common cancers affecting women worldwide [1]. Early detection and intervention of cervical lesions can effectively inhibit the occurrence and development of cervical cancer, which means a better prognosis [2]. Human papillomavirus infection is considered to be the leading cause of cervical cancer [3]. Persistent HR-HPV infection has been identified as a key role in the development of cervical cancer, with HPV-16 and HPV-18 being the most common genotypes infected in invasive cervical cancer [4].

Cytological lesion detection and HR-HPV detection are the two main methods to effectively screen cervical cancer and precancerous lesions [5]. Various HPV subtypes have different pathogenicity for cervical cancer. Can multiple HPV subtypes be used to predict cervical cytology? Our study analyzed the HPV infection based on different cytological results, to find out the specific HPV genotype that is more likely to cause cervical lesions, and probed into the possibility of predicting cervical cytological lesions with HPV genotype combinations.

## Materials and methods

### Participants

From January 2004 to December 2010, the study was carried out in the Sun Yat-Sen Memorial Hospital, Sun Yat-Sen University. Cytology samples were collected from 26,455 women that came to our hospital’s outpatient clinic for genital tract disease or routine cytological screening. Two thousand two hundred twenty-four women who received the HPV genotype examination at the same time were enrolled in this study.

### Liquid-based cytology test (LCT) and pathological diagnosis

Eligible patients received gynecological examinations performed by gynecological practitioners from Sun Yat-Sen Memorial Hospital. Samples of exfoliated cervical cells were collected using plastic cervical swabs during the examination. Insert a plastic cervical swab into the endocervical canal for 1–1.5 cm, and rotate it counterclockwise for 4–5 full circles. Immediately place the tip containing the cellular material into the conveying medium tube and store it at 4 °C. All store bottles with cellular materials were collected, mixed with 3 mL specimen stored liquid (Hybribio Biotechnology Limited Corp, Chaozhou, China) and stored in 4 °C. Using the Bethesda system to evaluate the result. The evaluation system included: (1) negative (A0), (2) atypical squamous cells (ASC), (3) low-grade squamous intraepithelial lesion (LSIL), (4) high-grade squamous intraepithelial lesion (HSIL), (5) squamous cell carcinoma (SCC), (6) abnormal glandular lesions (AGC, AIS), (7) atypical glandular cells of undetermined significance (AGCUS) and (8) adenocarcinoma.

### HPV detection and genotyping

Genotype detection (Hybribio, Ltd., Hong Kong) [6] is a PCR- based assay and can amplify 21 HPV genotypes, including 13 HR-HPV genotypes (16, 18, 31, 33, 35, 39, 45, 51, 52, 56, 58, 59, 68). The assay was performed according to manufacturer’s protocol. Briefly, after PCR amplification, the amplicons were subjected for hybridization. The assay utilized a “flow-through hybridization” technique by actively directing the targeting molecules toward the immobilized probes within the membrane fibers, and the complementary molecules were retained by forming duplexes. The hybrids were then detected by the addition of streptavidin- horseradish peroxidase conjugate and a substrate (NBT/BCIP).

### Statistical analysis

First, descriptive statistics were conducted, and then chi-square test (categorical data) and t test (continuous data) were used to evaluate the correlation between Pap testing and covariates. In order to test the existence of collinearity and interaction, relationships between all covariates were evaluated through the cross-tabulations of categorical variables and the correlation matrices of continuous variables. Unconditional logistic regression was used to analyze the potential association between HPV infection and cervical lesions. Odds ratio (OR) and 95% confidence intervals (95% CIs) were calculated. Data management and statistical analysis were performed with SPSS software version 16. *P* values were two-sided, and statistical significance was accepted if the P value was 0.05 or less.

## Results

A total of 2224 females were included in this study, with a mean age of 37.6 years (±9.2 SD), ranging from 21 to 77 years. Patients were admitted for post-coital bleeding, postmenopausal bleeding, last abnormal LCT, abnormal menstruation, irregular bleeding, and uterine prolapse (Table 1). All of the women were tested for liquid-based cytology and for the HPV genotype examination. Cytological abnormalities were found in 479 subjects. ASC-US (264, 11.9%) was found to be the most common cytological abnormalities, followed by LSIL (152, 6.8%). Women with the cytological results of SCC were older than those with other cytological findings. In addition, the mean age of patients with HSIL and ASC-H is higher than that of normal cytology patients (Table 1). HPV infection was detected in 688(30.9%) patients (including HR-HPV infection and LR-HPV infection). The HR-HPV infection rate was 27.8% (619/2224), and the LR-HPV infection rate was 5.0% (112/2224). Forty-three women were detected to have both HR-HPV and LR-HPV infections, and 135 women were detected with multiple HR-HPV infections. Furthermore, patients with cytological findings of ASC-US, LSIL, HSIL and SCC showed higher HPV positive rates and higher multiple HR-HPV positive rates than the normal and AGC results (Table 1).

**Table 1:** LCT detection results distribution

		normal	AGC	ASC-H	ASC-US	LSIL	HSIL	SCC
HPV status	N	1745 (78.5%)	8 (0.4%)	26 (1.2%)	264 (11.9%)	152 (6.8%)	23 (1.0%)	6 (0.3%)
	Age	37.9 ± 9.1	39.4 ± 14.5	40.1 ± 9.1	35.0 ± 9.2	36.6 ± 9.4	41.1 ± 7.8	44.8 ± 14.2
	HPV infection	359 (20.6%)	3 (37.5%)	19 (73.1%)	159 (60.2%)	127 (83.6%)	17 (73.9%)	4 (66.7%)
	HR-HPV infection	318 (18.2%)	3 (37.5%)	19 (73.1%)	140 (53.0%)	119 (78.3%)	16 (69.6%)	4 (66.7%)
	LR-HPV infection	60 (3.4%)	0 (0.0%)	3 (11.5%)	33 (12.5%)	15 (9.9%)	1 (4.3%)	0 (0.0%)
	HR + LR infection	19 (1.1%)	0 (0.0%)	3 (11.5%)	14 (5.3%)	7 (4.6%)	0 (0.0%)	0 (0.0%)
	Multiple HR-HPV infection	57 (3.3%)	0 (0.0%)	1 (3.8%)	33 (12.5%)	42 (27.6%)	2 (8.7%)	0 (0.0%)
Symptoms	Post-coital bleeding	238 (13.6%)	1 (12.5%)	5 (19.2%)	37 (14.0%)	30 (19.7%)	1 (4.3%)	1 (16.7%)
	Postmenopausal bleeding	493 (28.3%)	2 (25.0%)	3 (11.5%)	87 (33.0%)	51 (33.6%)	5 (21.7%)	2 (33.3%)
	Last Abnormal LCT	136 (7.8%)	2 (25.0%)	1 (3.8%)	29 (11.0%)	10 (6.6%)	0 (0.0%)	0 (0.0%)
	Abnormal menstruation	444 (25.4%)	1 (12.5%)	7 (26.9%)	67 (25.4%)	41 (27.0%)	7 (30.4%)	1 (16.7%)
	Irregular bleeding	72 (4.1%)	0 (0.0%)	1 (3.8%)	10 (3.8%)	9 (5.9%)	0 (0.0%)	0 (0.0%)
	Uterine prolapse	158 (9.1%)	1 (12.5%)	3 (11.5%)	26 (9.8%)	12 (7.9%)	5 (21.7%)	0 (0.0%)
	Others	589 (33.8%)	3 (37.5%)	13 (50.0%)	82 (31.1%)	42 (27.6%)	10 (43.5%)	2 (33.3%)

*LCT* Liquid-based cytology test

*HR-HPV* High-risk HPV

*LR-HPV* Low-risk HPV

The prevalence of HPV genotype was shown in Table 2. HPV-52(4.9%) was found to be the most common genotype in total, followed by HPV-16(4.2%), HPV-58(2.9%)and HPV-53(1.9%). The HPV infection rate in patients with different cytological examination results was different. As shown in Tables 3 & 4, HPV infection rate was significantly higher in cytology with ASC-US or worse patients (LCT+) than that of negative patients, except for HPV-35, HPV-43 and HPV-44. In patients with cytological results of HSIL and higher lesions (HSIL+), the infection rates of HPV-16, HPV-31, HPV-33, and HPV-58 were significantly higher than that of those patients with cytological results of LSIL and lower lesions (LSIL-).

**Table 2:** HPV genotype distribution in different LCT results

HPV genotype	Normal	AGC	ASC-H	ASC-US	LSIL	HSIL	SCC	Total
HPV-16	74 (4.2%)	3 (37.5%)	10 (38.5%)	27 (10.2%)	24 (15.8%)	6 (26.1%)	3 (50.0%)	147 (6.6%)
HPV-18	28 (1.6%)	0 (0.0%)	0 (0.0%)	13 (4.9%)	7 (4.6%)	0 (0.0%)	0 (0.0%)	48 (2.2%)
HPV-31	14 (0.8%)	0 (0.0%)	0 (0.0%)	7 (2.7%)	2 (1.3%)	2 (8.7%)	0 (0.0%)	25 (1.1%)
HPV-33	23 (1.3%)	0 (0.0%)	1 (3.8%)	3 (1.1%)	13 (8.6%)	4 (17.4%)	0 (0.0%)	44 (2.0%)
HPV-35	2 (0.1%)	0 (0.0%)	0 (0.0%)	0 (0.0%)	3 (2.0%)	0 (0.0%)	0 (0.0%)	5 (0.2%)
HPV-39	24 (1.4%)	0 (0.0%)	0 (0.0%)	12 (4.5%)	8 (5.3%)	0 (0.0%)	0 (0.0%)	44 (2.0%)
HPV-45	1 (0.1%)	0 (0.0%)	0 (0.0%)	1 (0.4%)	4 (2.6%)	0 (0.0%)	0 (0.0%)	6 (0.3%)
HPV-51	9 (0.5%)	0 (0.0%)	0 (0.0%)	5 (1.9%)	7 (4.6%)	0 (0.0%)	0 (0.0%)	21 (0.9%)
HPV-52	85 (4.9%)	0 (0.0%)	3 (11.5%)	44 (16.7%)	32 (21.1%)	1 (4.3%)	0 (0.0%)	165 (7.4%)
HPV-53	34 (1.9%)	0 (0.0%)	0 (0.0%)	18 (6.8%)	16 (10.5%)	0 (0.0%)	0 (0.0%)	68 (3.1%)
HPV-56	7 (0.4%)	0 (0.0%)	0 (0.0%)	2 (0.8%)	12 (7.9%)	0 (0.0%)	0 (0.0%)	21 (0.9%)
HPV-58	50 (2.9%)	0 (0.0%)	5 (19.2%)	25 (9.5%)	19 (12.5%)	5 (21.7%)	1 (16.7%)	105 (4.7%)
HPV-59	7 (0.4%)	0 (0.0%)	0 (0.0%)	8 (3.0%)	4 (2.6%)	0 (0.0%)	0 (0.0%)	19 (0.9%)
HPV-66	10 (0.6%)	0 (0.0%)	0 (0.0%)	4 (1.5%)	13 (8.6%)	0 (0.0%)	0 (0.0%)	27 (1.2%)
HPV-68	21 (1.2%)	0 (0.0%)	1 (3.8%)	11 (4.2%)	5 (3.3%)	0 (0.0%)	0 (0.0%)	38 (1.7%)
HPV-6	11 (0.6%)	0 (0.0%)	0 (0.0%)	7 (2.7%)	1 (0.7%)	1 (4.3%)	0 (0.0%)	20 (0.9%)
HPV-11	12 (0.7%)	0 (0.0%)	1 (3.8%)	10 (3.8%)	2 (1.3%)	0 (0.0%)	0 (0.0%)	25 (1.1%)
HPV-42	1 (0.1%)	0 (0.0%)	0 (0.0%)	3 (1.1%)	0 (0.0%)	0 (0.0%)	0 (0.0%)	4 (0.2%)
HPV-43	2 (0.1%)	0 (0.0%)	0 (0.0%)	0 (0.0%)	1 (0.7%)	0 (0.0%)	0 (0.0%)	3 (0.1%)
HPV-44	8 (0.5%)	0 (0.0%)	0 (0.0%)	1 (0.4%)	3 (2.0%)	0 (0.0%)	0 (0.0%)	12 (0.5%)
cp8304	28 (1.6%)	0 (0.0%)	2 (7.7%)	15 (5.7%)	9 (5.9%)	0 (0.0%)	0 (0.0%)	54 (2.4%)

**Table 3:** HPV-specific risks for LCT results

Genotypes	LCT (−)	LCT (+)	P	OR (for LCT (+))	95%CI
HPV-16	74 (4.2%)	73 (15.2%)	< 0.001	4.06	2.89–5.71
HPV-18	28 (1.6%)	20 (4.2%)	0.001	2.672	1.49–4.79
HPV-31	14 (0.8%)	11 (2.3%)	0.009	2.906	1.31–6.44
HPV-33	23 (1.3%)	21 (4.4%)	< 0.001	3.433	1.88–6.26
HPV-35	2 (0.1%)	3 (0.6%)	0.062	5.493	0.92–32.97
HPV-39	24 (1.4%)	20 (4.2%)	< 0.001	3.125	1.71–5.71
HPV-45	1 (0.1%)	5 (1.0%)	0.008	18.397	2.14–157.84
HPV-51	9 (0.5%)	12 (2.5%)	< 0.001	4.956	2.08–11.83
HPV-52	85 (4.9%)	80 (16.7%)	< 0.001	3.916	2.83–5.42
HPV-53	34 (1.9%)	34 (7.1%)	< 0.001	3.845	2.36–6.26
HPV-56	7 (0.4%)	14 (2.9%)	< 0.001	7.475	3.00–18.63
HPV-58	50 (2.9%)	55 (11.5%)	< 0.001	4.397	2.96–6.54
HPV-59	7 (0.4%)	12 (2.5%)	< 0.001	6.38	2.50–16.30
HPV-66	10 (0.6%)	17 (3.5%)	< 0.001	6.384	2.90–14.04
HPV-68	21 (1.2%)	17 (3.5%)	0.001	3.021	1.58–5.77
HPV-6	11 (0.6%)	9 (1.9%)	0.015	3.019	1.24–7.33
HPV-11	12 (0.7%)	13 (2.7%)	0.001	4.029	1.83–8.89
HPV-42	1 (0.1%)	3 (0.6%)	0.038	10.992	1.14–105.91
HPV-43	2 (0.1%)	1 (0.2%)	0.624	1.823	0.17–20.15
HPV-44	8 (0.5%)	4 (0.8%)	0.326	1.828	0.55–6.10
cp8304	28 (1.6%)	26 (5.4%)	< 0.001	3.52	2.04–6.06
multiple HPV infection	72 (4.1%)	100 (20.9%)	< 0.001	6.131	4.439–8.467

*LCT (−)* Normal LCT results

*LCT (+)* Cytology with ASC-US or worse

OR: odds ratio

**Table 4:** HPV-specific risks for HSIL and higher lesions

HPV genotype	LSIL-	HSIL+	P	OR (for HSIL+)	95%CI
HPV-16	138 (6.3%)	9 (31.0%)	< 0.001	6.708	3.00–15.01
HPV-31	23 (1.0%)	2 (6.9%)	0.011	6.995	1.57–31.16
HPV-33	40 (1.8%)	4 (13.8%)	< 0.001	8.62	2.87–25.92
HPV-52	164 (7.5%)	1 (3.4%)	0.424	0.442	0.06–3.27
HPV-58	99 (4.5%)	6 (20.7%)	< 0.001	5.523	2.20–13.87
HPV-6	19 (0.9%)	1 (3.4%)	0.177	4.09	0.53–31.62
multiple HPV infection	170 (7.7%)	2 (6.9%)	0.865	0.882	0.208–3.742

*LSIL-* Including normal, AGC, ASC-H, ASC-US and LSIL

*HSIL+* Including HSIL and SCC

*OR* Odds ratio

Multivariate logistic regression was used to explore the relationship between different HPV subtypes of infection and cytological abnormalities, and to adjust the effect of age on cell lesions. As shown in Table 5, multivariate logistic regression shows HPV-16, 18, 33, 39, 45, 51, 52, 53, 56, 58, 59, 66, 68, 11, 42 and CP8304 were significantly associated with positive correlation LCT results. In addition, we used multivariate regression to analyze the relationship between different HPV subtypes and HSIL+ lesions, HPV-16, 31, 33, and 58 were shown independent predictors for HSIL and above cytological abnormalities.

**Table 5:** Multiple logistic regression analysis for LCT

Genotypes	P	OR (for LCT (+))	95%CI
HPV-16	< 0.001	4.591	3.17–6.66
HPV-18	0.036	2.021	1.05–3.90
HPV-33	0.001	3.165	1.61–6.22
HPV-39	0.002	2.841	1.47–5.50
HPV-45	0.025	13.775	1.40–135.92
HPV-51	0.002	4.765	1.80–12.63
HPV-52	< 0.001	3.623	2.54–5.17
HPV-53	< 0.001	3.796	2.20–6.54
HPV-56	0.001	5.747	2.13–15.52
HPV-58	< 0.001	5.034	3.27–7.76
HPV-59	0.001	5.838	2.11–16.13
HPV-66	< 0.001	6.001	2.55–14.14
HPV-68	0.027	2.299	1.10–4.80
HPV-11	0.009	3.21	1.34–7.70
HPV-42	0.039	11.811	1.14–122.64
cp8304	< 0.001	2.984	1.62–5.51
age			
21–30	0.001	1	
31–40	0.015	1.727	1.11–2.69
41–50	0.736	1.076	0.70–1.65
> 50	0.915	0.975	0.62–1.54

*LCT (+)* Positive LCT results

*OR* Odds ratio

**Fig. 1:**
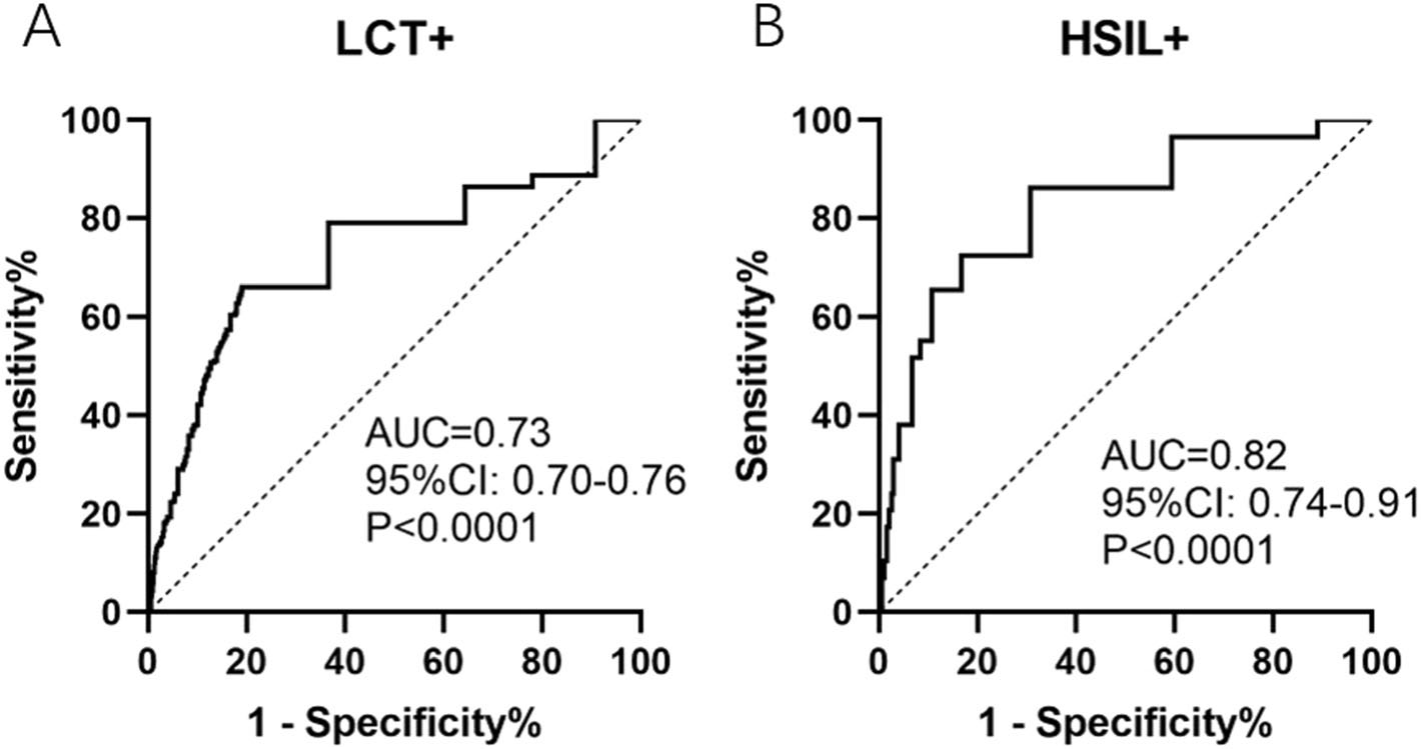
ROC curve was used to evaluate the prediction efficiency of the LCT results. **A** ROC curve for positive LCT results; **B** ROC for HSIL and SCC

On the basis of the above results, we established a prediction model to predict cervical cytology, and calculated the corresponding risk indicators. A Receiver Operating Characteristic curve (ROC) was used to evaluate the prediction efficiency. The results show that the model has a good predictive effect on cell lesions, and the area under the ROC curve (AUC) is equal to 0.73 (Fig. 1A). Additionally, the prediction efficiency for HSIL+ shows an AUC of 0.82 (Fig. 1B).

## Discussion

This study analyzed the risk of HPV infection in different cervical lesions and the risk of different cervical lesions in the case of HPV infection. HPV infection is considered to be the main risk factor for cervical cancer pathologies, particularly for types 16 and 18, which are associated with approximately 70% of cervical cancers [7]. According to our study, HPV-52 was found to be the most common genotype in total, which is inconsistent with previous studies [8, 9], but similar to previous studies in Gambia, Kenya and Tanzania [10–14]. This difference may be due to regional and ethnic differences, as well as to the bias of selecting only in-patient examinations. In patients with abnormal cytological findings, HPV infection rates were higher than normal, consistent with previous studies showing a strong association between cervical lesions and HPV infection [4]. Meanwhile, high positive rates of HPV-16,31,33,58 in HSIL+ lesion patients also supported the association between persistent HR-HPV infection and high-grade cervical lesions [15, 16]. The result was consistent with previous studies, which showed that alpha-9 species of HR-HPV types were more likely to develop persistent infection and lead to serious cervical lesions [17].

In order to investigate prediction value of specific genotype HPV infection for cervical lesions, the multivariate logistic-regression analysis was performed and suggests that HPV genotype infection is independently associated with cytological abnormalities. Using our multivariate logistic-regression model, combined HPV subtypes have a good predictive potential for predicting cytological abnormalities. Cytology combined with HR-HPV detection was recommended for cervical cancer screening by US Preventive Services Task Force [5]. Whereas, if the combined HPV test can effectively predict cytological abnormalities, it will be possible to use the combined HPV test alone for cervical cancer screening.

The advantage of our research is that, as far as we know, there is no research on the use of HPV genotype testing to predict cervical cytology, and we are trying to fill the knowledge gap by using multivariable logistic regression analysis. However, there are some potential limitations to our research. First, the prediction model has not been externally verified, so its validity needs further discussion. On the other hand, it is not clear whether the retrospective analysis of the data from the study, which was carried out on the patients who came to the clinic or underwent a physical examination, had the same high predictive effect for all women. Finally, in the absence of corresponding histological results, the feasibility of substituting the results of human papillomavirus tests for cytological tests deserves further study.

In summary, our study suggested that HPV-16,31,33 and 58 infections were significant risk factors for cervical lesions. Combined HPV genotypes test can effectively predict cytological abnormalities.

## Data Availability

The data that support the findings of this study are available from Sun Yat-sen Memorial Hospital but restrictions apply to the availability of these data, which were used under license for the current study, and so are not publicly available. Data are however available from the first author upon reasonable request and with permission of Sun Yat-sen Memorial Hospital.

## Abbreviations

SCC: Squamous cervical cancer
CI: Confidence interval
HPV: Human papillomavirus
HR-HPV: High-risk HPV
LR-HPV: Low-risk HPV
HSIL: High-grade squamous intraepithelial lesions
LCT: Liquid-based cytology test
LSIL: Low-grade squamous intraepithelial lesions
OR: Odds ratio

## References

[1] Torre LA, Islami F, Siegel RL, Ward EM, Jemal A (2017). Global Cancer in women: burden and trends. Cancer Epidemiol Biomark Prev.

[2] Vaccarella S, Laversanne M, Ferlay J, Bray F (2017). Cervical cancer in Africa, Latin America and the Caribbean and Asia: regional inequalities and changing trends. Int J Cancer.

[3] Appleby P (2007). Cervical cancer and hormonal contraceptives: collaborative reanalysis of individual data for 16,573 women with cervical cancer and 35,509 women without cervical cancer from 24 epidemiological studies. Lancet.

[4] de Sanjose S, Quint WG, Alemany L, Geraets DT, Klaustermeier JE, Lloveras B, Tous S, Felix A, Bravo LE, Shin HR, Vallejos CS, de Ruiz PA, Lima MA, Guimera N, Clavero O, Alejo M, Llombart-Bosch A, Cheng-Yang C, Tatti SA, Kasamatsu E, Iljazovic E, Odida M, Prado R, Seoud M, Grce M, Usubutun A, Jain A, Suarez GA, Lombardi LE, Banjo A, Menéndez C, Domingo EJ, Velasco J, Nessa A, Chichareon SC, Qiao YL, Lerma E, Garland SM, Sasagawa T, Ferrera A, Hammouda D, Mariani L, Pelayo A, Steiner I, Oliva E, Meijer CJ, al-Jassar WF, Cruz E, Wright TC, Puras A, Llave CL, Tzardi M, Agorastos T, Garcia-Barriola V, Clavel C, Ordi J, Andújar M, Castellsagué X, Sánchez GI, Nowakowski AM, Bornstein J, Muñoz N, Bosch FX, Retrospective International Survey and HPV Time Trends Study Group (2010). Human papillomavirus genotype attribution in invasive cervical cancer: a retrospective cross-sectional worldwide study. Lancet Oncol.

[5] Curry SJ (2018). Screening for cervical Cancer: US preventive services task force recommendation statement. Jama.

[6] Liu SS, Leung RCY, Chan KKL, Cheung ANY, Ngan HYS (2010). Evaluation of a newly developed GenoArray human papillomavirus (HPV) genotyping assay and comparison with the Roche linear Array HPV genotyping assay. J Clin Microbiol.

[7] Walboomers JM (1999). Human papillomavirus is a necessary cause of invasive cervical cancer worldwide. J Pathol.

[8] Guan P, Howell-Jones R, Li N, Bruni L, de Sanjosé S, Franceschi S, Clifford GM (2012). Human papillomavirus types in 115,789 HPV-positive women: a meta-analysis from cervical infection to cancer. Int J Cancer.

[9] Chen HC, Schiffman M, Lin CY, Pan MH, You SL, Chuang LC, Hsieh CY, Liaw KL, Hsing AW, Chen CJ, for the CBCSP-HPV Study Group (2011). Persistence of type-specific human papillomavirus infection and increased long-term risk of cervical cancer. J Natl Cancer Inst.

[10] Mbaye el HS (2014). Human papillomavirus infection in women in four regions of Senegal. J Med Virol.

[11] De Vuyst H (2003). Distribution of human papillomavirus in a family planning population in Nairobi, Kenya. Sex Transm Dis.

[12] Dartell M, Rasch V, Kahesa C, Mwaiselage J, Ngoma T, Junge J, Gernow A, Funch Ejlersen S, Munk C, Iftner T, Kjaer SK (2012). Human papillomavirus prevalence and type distribution in 3603 HIV-positive and HIV-negative women in the general population of Tanzania: the PROTECT study. Sex Transm Dis.

[13] Rahman M, Sasagawa T, Yamada R, Kingoro A, Ichimura H, Makinoda S (2011). High prevalence of intermediate-risk human papillomavirus infection in uterine cervices of Kenyan women infected with human immunodeficiency virus. J Med Virol.

[14] Bruni L, Diaz M, Castellsagué X, Ferrer E, Bosch FX, de Sanjosé S (2010). Cervical human papillomavirus prevalence in 5 continents: meta-analysis of 1 million women with normal cytological findings. J Infect Dis.

[15] Ho GY (1998). Natural history of cervicovaginal papillomavirus infection in young women. N Engl J Med.

[16] Zur Hausen H (2002). Papillomaviruses and cancer: from basic studies to clinical application. Nat Rev Cancer.

[17] Kjaer SK, Frederiksen K, Munk C, Iftner T (2010). Long-term absolute risk of cervical intraepithelial neoplasia grade 3 or worse following human papillomavirus infection: role of persistence. J Natl Cancer Inst.

